# Construction, Visualisation, and Clustering of Transcription Networks from Microarray Expression Data

**DOI:** 10.1371/journal.pcbi.0030206

**Published:** 2007-10-26

**Authors:** Tom C Freeman, Leon Goldovsky, Markus Brosch, Stijn van Dongen, Pierre Mazière, Russell J Grocock, Shiri Freilich, Janet Thornton, Anton J Enright

**Affiliations:** 1 Division of Pathway Medicine, University of Edinburgh Medical School, Edinburgh, United Kingdom; 2 Wellcome Trust Sanger Institute, Hinxton, Cambridge, United Kingdom; 3 European Bioinformatics Institute, Hinxton, Cambridge, United Kingdom; University of Manchester, United Kingdom

## Abstract

Network analysis transcends conventional pairwise approaches to data analysis as the context of components in a network graph can be taken into account. Such approaches are increasingly being applied to genomics data, where functional linkages are used to connect genes or proteins. However, while microarray gene expression datasets are now abundant and of high quality, few approaches have been developed for analysis of such data in a network context. We present a novel approach for 3-D visualisation and analysis of transcriptional networks generated from microarray data. These networks consist of nodes representing transcripts connected by virtue of their expression profile similarity across multiple conditions. Analysing genome-wide gene transcription across 61 mouse tissues, we describe the unusual topography of the large and highly structured networks produced, and demonstrate how they can be used to visualise, cluster, and mine large datasets. This approach is fast, intuitive, and versatile, and allows the identification of biological relationships that may be missed by conventional analysis techniques. This work has been implemented in a freely available open-source application named BioLayout *Express* ^3D^.

## Author Summary

This paper describes a novel approach for analysis of gene expression data. In this approach, normalized gene expression data is transformed into a graph where nodes in the graph represent transcripts connected to each other by virtue of their coexpression across multiple tissues or samples. The graph paradigm has many advantages for such analyses. Graph clustering of the derived network performs extremely well in comparison to traditional pairwise schemes. We show that this approach is robust and able to accommodate large datasets such as the Genomics Institute of the Novartis Research Foundation mouse tissue atlas. The entire approach and algorithms are combined into a single open-source JAVA application that allows users to perform this analysis and further mining on their own data and to visualize the results interactively in 3-D. The approach is not limited to gene expression data but would also be useful for other complex biological datasets. We use the method to investigate the relationship between the phylogenetic age of transcripts and their tissue specificity.

## Introduction

Complete genome sequencing of hundreds of pathogenic and model organisms has provided the parts list required for large-scale studies of gene function [1]. Enormous amounts of data pertaining to the nature of genes and proteins and their interactions in the cell have now been generated by techniques including, but not limited to: gene coexpression analysis, yeast two-hybrid assays, mass spectrometry, and RNA interference [2]. Such functional genomics and proteomics approaches, when combined with computational biology and the emerging discipline of systems biology, finally allow us to begin comprehensive mapping of cellular and molecular networks and pathways [3,4]. One of the main difficulties we currently face is how best to integrate these disparate data sources and use them to better understand biological systems in health and disease [5].

Visualisation and analysis of biological data as networks is becoming an increasingly important approach to explore a wide variety of biological relationships. Such approaches have already been used successfully in the study of sequence similarity, protein structure, protein interactions, and evolution [6–8]. Shifting biological data into a graph/network paradigm allows one to use algorithms, techniques, ideas, and statistics previously developed in graph theory, engineering, computer science, and (more recently) computational systems biology. In classical graph theory, a graph or network consists of *nodes* connected by *edges*. For biological networks, nodes are usually genes, transcripts, or proteins, while edges tend to represent experimentally determined similarities or functional linkages between them [9].

Conventional analysis techniques are generally pairwise, where an individual relationship between two biological entities is studied without considering higher-order interactions with their neighbours. Graph and network analysis techniques allow the exploration of the position of a biological entity in the context of its local neighbourhood in the graph, and the network as a whole [7]. Another important advantage of such techniques is that for noisy datasets, spurious edges tend not to form structure (or cliques) in the resultant graph, but instead randomly link nodes; although this may not be the case for data generated by techniques with inherent technical biases. Because many network analysis techniques (e.g., graph clustering) exploit local structure in networks between biologically related nodes, they are far less troubled by inherent noise, which may confound conventional pairwise approaches [7].

One example of network analysis is the clustering of protein–protein similarity and interaction networks. These techniques illustrate that graph clustering (i.e., clustering genes/proteins with respect to their graph context, rather than iterative pairwise clustering) performs extremely well and allows the discovery of novel aspects of biological function [7]. Such techniques can hence provide insight into both local features of networks (e.g., prediction of pathways or functional modules) and also global features of the network (e.g., small-world behaviour and centrality [10–12]).

Although network analysis of biological data has shown great promise, little attention has been paid to microarray gene expression data. These data are now abundant, generally of high quality, and consist of the type of high-dimensional data for which such approaches are well-suited. In principle, transformation of gene expression data into a network graph holds few challenges. The similarity between individual expression profiles may be determined by one of a number of possible statistical techniques, e.g., the Pearson and Spearman correlation coefficients [13]. Networks can be constructed by connecting transcripts (nodes) by edges that infer varying degrees of coexpression based on an arbitrary correlation threshold [14]. Indeed, a number of groups have previously sought to apply the network paradigm to microarray data, establishing relationships between genes based on correlation of expression [14–18]. While these studies have suggested the power of this approach, limitations in the functionality and visualisation capabilities of the tools supporting their attempts have severely limited their approaches for general application.

In this manuscript, we describe the development and application of a new network analysis tool, BioLayout *Express* ^3D^, that facilitates the construction, visualisation, clustering, and analysis of microarray gene expression data. Specifically, we chose to analyse the Genomics Institute of the Novartis Research Foundation (GNF) mouse tissue gene expression atlas to demonstrate the efficacy of this approach [19]. The GNF data was generated so as to provide a genome-wide analysis of transcript abundance across a wide range of normal tissue and cell types. This dataset represents one of the most complete systematic studies of tissue-specific expression in the mammalian transcriptome to date. However, in common with other large datasets, analysis of these data presents significant challenges. Certain genes are known to only be expressed by a single cell type, at specific times during development, or in response to explicit stimuli. Others are thought to be expressed by all cells simultaneously at about the same level. Between these two extremes, there are many other genes that are expressed in most or a number of cell types, but whose transcription may be regulated to give a specific temporal and spatial pattern of expression. It is also known that genes that play distinct roles in a common pathway or biological process are often expressed in a similar manner; i.e., they are coexpressed [20]. Hence, when genes are found to have analogous expression profiles, this may indicate that the genes have linked functional activities. To better understand aspects of gene regulation and the functional role of the encoded proteins, we chose to explore the utility of network analysis to explore the innate structure of this dataset. We demonstrate that this approach can accurately locate clusters of genes sharing similar network connectivity (expression pattern), the relationships between these clusters, and statistical analysis of functional annotations.

## Results

### Generation of Networks from Microarray Expression Data

**Figure 1:**
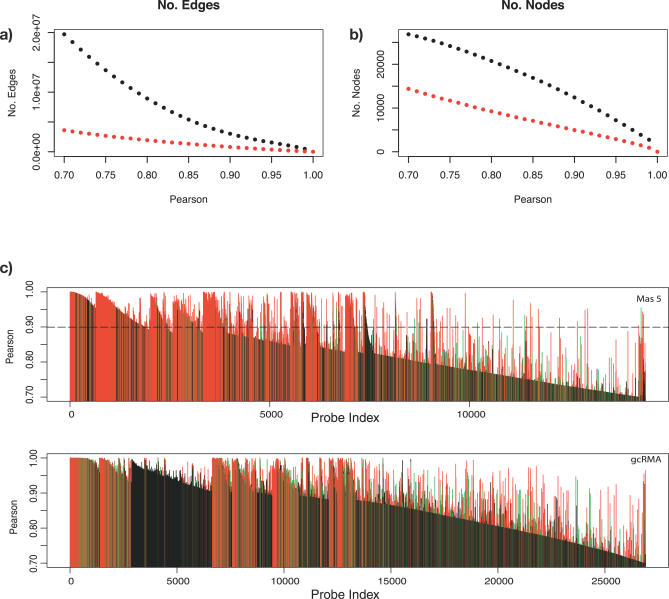
Relationship between Pearson Correlation Coefficient of Expression Profiles and Node Inclusion into Networks As the Pearson threshold decreases, the number of (nonsingleton) nodes (A) and edges connecting these nodes (B) increases, resulting in larger networks. The red dotted lines show this relationship for MAS5 scaled data, and black dotted lines show gcRMA normalised data. “Landscape” plots (C–D) also demonstrate the inherent structure within the data and the effect of different normalisation methods. All probe-set pairs with a Pearson correlation coefficient ≥0.7 have been plotted, each probe-set joining the graph nearest to the probe-set(s) to which it shares this relationship. The resultant “landscape” shows a number of peaks corresponding to groups of genes sharing similar expression profiles. The data corresponding to individual probe-sets have been coloured according to the maximum signal across all samples: red denotes the top third of transcripts with the highest maximum expression; green, the middle third, and black, the lowest third. The dashed black line (C) denotes the Pearson threshold used for subsequent analyses.

Generation of network graphs from gene expression data uses the Pearson correlation coefficient as a measure of similarity between expression profiles. An expression profile is defined as the data derived from the range of samples analysed, originating from an individual probe (-set). Pairwise Pearson correlation coefficients were calculated for every probe-set on the array and correlation coefficients above a predefined threshold used to draw edges between genes (nodes) in the construction of network graphs (see Methods). Without imposing a correlation threshold, every node would be connected to every other with a weighted edge (−1.0 ≥ *p* ≤ 1.0). Obviously, the resulting graphs would be overly large and dominated by uninformative edges. Hence, this initial thresholding is used to define a starting point for subsequent analysis, and nodes (probes) with no connections above the selected threshold are removed from the graph. The size of the graph produced is therefore dependent on the threshold level selected. At low Pearson correlation coefficient cutoffs (*p* ≤ 0.8), graphs are (too) large with many nodes and edges (Figure 1A and 1B). At higher thresholds, the networks consist of a smaller number of genes and tend to be more useful for most analyses. This relationship is illustrated using a “landscape” plot that we have developed, which provides a view of the innate structure within the GNF mouse atlas dataset (Figure 1C and 1D). This representation is a uniquely determined transformation of a dendrogram to a histogram (see Methods).

This representation is in many ways analogous to a dendrogram; however, here each bar in this plot represents a probe-set. The height of this bar corresponds to the Pearson correlation at which a probe first connects to another probe-set connected to network at a higher correlation coefficient. Hence, groups of genes that share high degrees of correlation in their expression appear as peaks within the landscape. A horizontal partitioning of the graph at a given Pearson threshold indicates those groups of genes that would form graphs above that threshold. Each disconnected peak above the Pearson threshold forms a separate graph.

These plots show that different normalisation strategies (gcRMA [21,22] and MAS5 [23,24]) markedly alter connectivity structures of the data and the characteristics of each network (Figure 1). It is interesting to observe that when the data corresponding to individual probe-sets are coloured according to the maximum signal across all samples, peaks are generally composed of genes that are highly expressed (red) in at least one sample. Indeed, almost all 16,104 probe-sets comprising the MAS5 landscape are drawn from data in the top two-thirds (red, green) of expressed data. In contrast, not only are there far more probe-sets included in the landscape of the gcRMA data (indicating a general increase in the connectivity of the data), but there is clearly more low-intensity data (black) being drawn into the graph. Quantile normalisation reduces the inherent randomness of low-intensity data, which increases its potential to form connections with other low-intensity data. Because of this and the fact that gcRMA assumes that all the data to be compared should have a similar distribution (which may well not be the case when comparing RNA from different tissues), we chose to concentrate on data normalised by MAS5 scaling.

### Characteristics of the Derived Network

**Figure 2:**
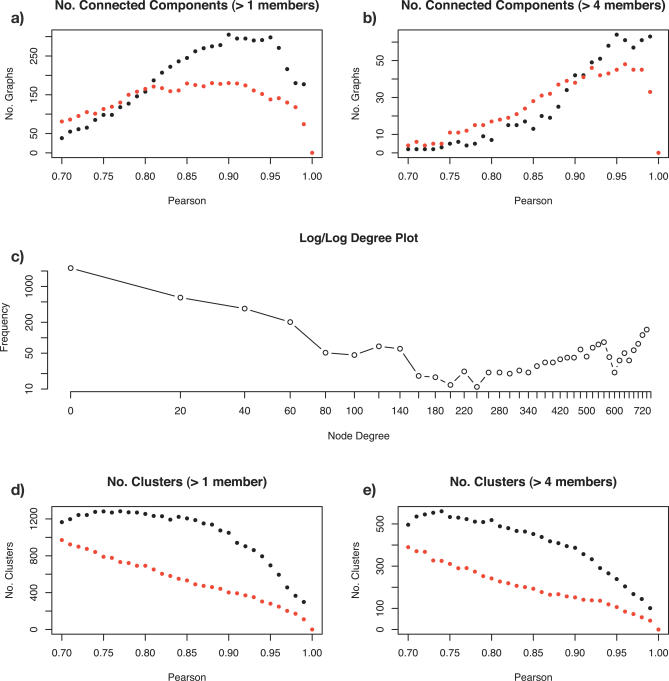
Network Connectivity and Clustering The relationships are shown (A–B) between the number of connected components in the GNF mouse tissue expression network and Pearson correlation coefficient threshold used. As the threshold increases, the tendency is for the network to fragment into smaller unconnected graphs. However, it can be seen from the difference between graphs (A–B) that many of these unconnected components comprise relatively few nodes. (C) Log–log frequency plot of node degree (i.e., total number of edges for each node) for the 0.9 Pearson threshold graph. These networks show an unusual topography relative to other networks derived from biological data. Here, a relatively large number of nodes show high-degree connectivity. These nodes represent genes forming core structures within the network being highly connected to neighbouring nodes. (D) MCL cluster counts (with inflation threshold set at 2.2) for networks derived at varying Pearson thresholds. Small clusters (≤4) account for a high proportion of the overall number of clusters (E). The red dotted lines show these relationships for MAS5 scaled data; the black dotted lines show gcRMA normalised data.

Figure 2A and 2B illustrate the relationship between the Pearson correlation threshold chosen and the number of nodes (probe-sets) and edges between them in the network graph(s) of the mouse transcriptome. Increasing the Pearson threshold removes edges and large networks fragment into multiple disconnected graphs (Figure 2A). A large proportion of these graphs consist of a small number of nodes (less than four) that could form by chance, but are also commonly formed between multiple probe-sets representing the same gene. Removal of small graphs aids interpretation of the data (Figure 2B). The connectivity, diameter, and other standard graph characteristics derived from the resulting networks are shown (Table 1).

**Table 1:**
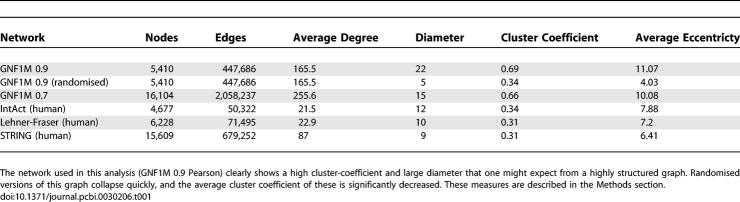
Various Measures of Graph Structure and Topology across the Mouse Atlas Network and Various Other Networks for Comparison

It is clear from these data that the network derived from the GNF1M mouse tissue atlas is highly structured (Table 1). Although many nodes have numerous connections (average degree = 165.5), the overall diameter of the network is large and the clustering coefficient is high (22 and 0.69, respectively; see Methods). These metrics indicate a highly structured graph amenable to graph-based analysis and clustering. This effect is distinctly nonrandom, as randomised networks (with the same degree and size) exhibit a far lower diameter and clustering coefficient (5 and 0.34). These results compare favourably with other networks that have been generated previously (IntAct, Lehner-Fraser, and STRING [35–37]). In fact, the gene expression graph has almost twice the diameter and clustering coefficient of these graphs. These results also hold when the GNF1M network is considered at 0.7 (Table 1). It is this innate structure in the graph that is exploited by the Markov cluster (MCL) algorithm for clustering.

The highly structured nature of this network illustrates that local network topology is primarily driven by biological relationships between gene expression across tissues. An example of network connectivity is shown (Figure 2C) derived at *p* ≥ 0.9. It is clear that a relatively large number of the nodes in the network have a high degree of connectivity. This is well above the number that might be expected if the network conformed to a linear power-law distribution. At lower Pearson thresholds, we may expect a large number of spurious edges. However, such edges tend to be added to the network in a random manner and are highly unlikely to form structured cliques in the derived network that could be mistakenly identified as biologically significant.

### Visualisation of the Network

**Figure 3:**
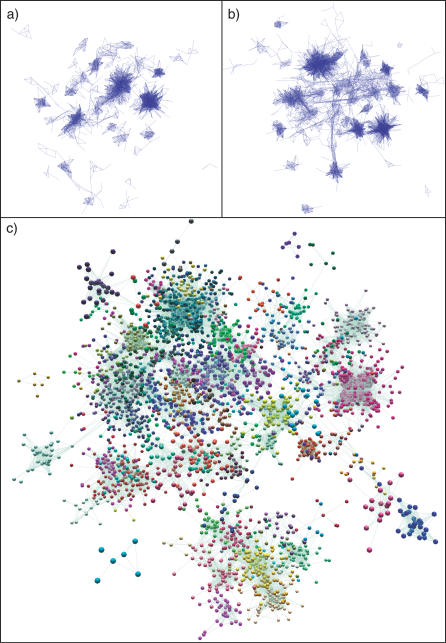
Untiled (Organic Layout) of GNF1M Network Graphs at Different Pearson Correlation Thresholds Graphs show the mouse tissue transcription network graphs when the Pearson threshold is a set at (A) 0.98 (1,421 nodes, 69,334 edges), (B) 0.95 (2,860 nodes, 201,724 edges), and (C) 0.90 (5,410 nodes, 447,467 edges). In graphs (A) and (B), nodes have been hidden so as to show the structure of the networks, and in (C) nodes are shown and coloured according to their membership of MCL clusters (inflation value 1.5).

The resulting graph (Figure 3) is a weighted nondirected graph consisting of probes connected by their coexpression values (*p* ≥ 0.7). Visual representation of such data is especially desirable, as it is far easier for humans to infer relationships and identify structural features from a visual standpoint. We use a highly optimised weighted Fruchterman-Rheingold layout algorithm [25] to quickly determine a reasonable layout of the network (see Methods). Due to the size and complexity of the resulting graphs, we performed this layout in 3-D and used the OpenGL [26] specification to allow us to render this complex 3-D graph interactively (see Methods). The resulting visualisation of the network allows for interactive analysis of very large networks (up to 10^5^ nodes and 10^6^ edges) using generally available hardware and accelerated graphics cards. This visualisation acts as a common interface for all of the subsequent analysis described below. Furthermore, the visualisation allows the user to quickly identify structures and features in the graph by eye that would not have been obvious previously. The approach also has the distinct advantage of using no prior assumptions as to the likely structure within the data (e.g., number of clusters) or questions to be addressed.

### MCL

We expect gene clusters (cliques of interconnected nodes) detected in this network to be biologically significant, identifying coexpressed genes likely to share regulatory mechanisms and functional relationships. Graph-clustering techniques are ideal for partitioning structured graphs into node clusters based on local neighbourhood topology [27]. Here we use the MCL algorithm [27] (see Methods). One distinct advantage of MCL is its ability to avoid incorrect clustering assignments in the presence of spurious edges (false positives). This is due to the fact that MCL discovers clusters by virtue of nodes sharing higher-order connectivity in their local neighbourhood and not merely pairwise linkages. Randomly placing edges in a graph may produce novel pairwise connections, but is unlikely to form ordered local structures (cliques) that would influence MCL clustering significantly.

To test this hypothesis, we devised an experiment in which we took the GNF1M 0.9 Pearson network and randomly added Gaussian noise to edge weights. The modified network was clustered using both MCL and a conventional single-linkage clustering algorithm to compare distances from their original clusterings. This shows (Figure S1) that even a small amount of noise (e.g., 0.01 standard deviation) produces a profound effect on single-linkage clustering (2%–4% deviation) but little effect on MCL clusters (0.1%–0.3%). Similarly, larger amounts of noise (e.g., 0.05) significantly disrupt (3%–13% deviation) single-linkage clusters with relatively little effect to MCL clusterings (0.3%–1.5% deviation). The MCL algorithm has also been shown to work exceptionally well for clustering structured graphs [7,28]. Below, we show that such expression graphs are indeed highly structured and, as such, are well-suited to MCL clustering. We believe these features further support the use of MCL clustering on transcription networks and represent an attractive alternative to conventional approaches.

### Structure of the Mouse Tissue Transcription Network and Biological Interpretation of Clusters

**Figure 4:**
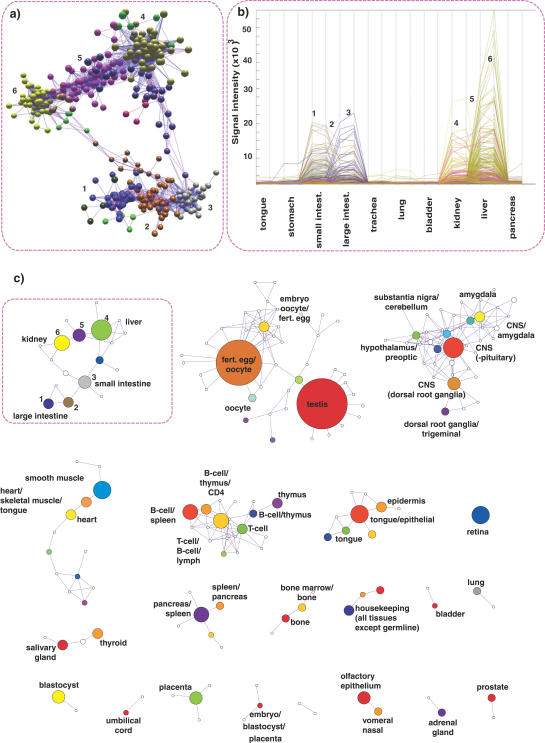
Tiled Graphs of the Network Formed from the GNF Mouse Tissue Data Using a 0.9 Pearson Correlation Threshold (A) For simplicity, the network is shown as collapsed MCL clusters (inflation value 2.2). A total of 33 disconnected graphs containing 168 clusters (≥4 nodes) are formed, the size (volume) of the node representing each cluster being proportional to the size of the cluster. (B) One graph extracted from a GNF mouse tissue network (highlighted in Figure 3A and demonstrates how individual nodes may be viewed in the context of the network neighbourhood). Plotting the average signal of the major node-clusterings in this graph over the tissues in which they are predominately expressed, one can see how expression profiles of the underlying genes change from one cluster to the next (C). Cluster 6 genes (yellow) for instance show a marked specificity for the kidney expression, whereas cluster 4 (green) genes are predominantly liver-specific. In the middle of these two clusters lies cluster 5 (purple), whose genes are expressed in both organs. The closer genes sit in the network to clusters 4 or 6, the stronger their expression will be in one tissue relative to the other. A similar relationship is true for small intestine (cluster 3)– and large intestine (clusters 1 and 2)–specific genes, with certain intestinally expressed genes also being expressed in the liver and kidney, connecting the expression networks of these two organ systems.

We explored the GNF1M expression data at a range of Pearson correlation thresholds and clustered at various MCL (v06–058) inflation values. We settled on a Pearson threshold of 0.9 and an MCL inflation value of 2.2 to fragment the data into biologically meaningful clusters (Figure 4). This was determined empirically based on maintaining a conservative balance between layout and clustering. Below this Pearson threshold, although new genes are added to the graph, they do not form any major new gene-dense network structures, instead being merely additions to, or forming bridges between, the structures already formed. A detailed view of a number of connected clusters is shown (Figure 4A) together with the expression data underlying their clustering (Figure 4B). To simplify this view across the whole network, it is possible to draw a cluster graph (Figure 4C) in which all nodes within a cluster are condensed into a single node representing that cluster whose volume reflects the number of members within each cluster. This view allows one to see how individual clusters are connected by virtue of their constituent nodes sharing coexpression across clusters.

All major tissues were found to have a certain number of genes expressed specifically within. It is interesting to note that by far the largest two clusters are associated with genes expressed predominately in the organs associated with gamete production (testis, 888 transcripts; and fertilised egg/oocyte, 753 transcripts; respectively). By contrast, the next largest cluster of liver-specific genes contains only 162 transcripts. Several major clusters appear to originate from genes expressed specifically, but across a number of tissues. These appear to originate from cell-specific genes expressed in cells performing a similar function in a number of tissues, e.g., keratins, myosins, etc. Indeed, it can be argued that all the genes present in this network represent cell-specific expression. However, when a number of genes are coexpressed specifically in the same tissue, it is impossible to know whether they are expressed in the same tissue-specific cell type.

The layout data in this manner has a number of distinct advantages. The position of each node (gene) within the network can be determined relative to its immediate neighbours; i.e., genes that are closest in expression (share edges) to that selected (Figure 4). Furthermore, the relationship between clusters can also be easily assessed. Indeed, adjoining clusters tend to share a degree of commonality in the expression of those genes that comprise them, but also have distinct differences. Figure 4A and 4B show the network of expression formed between regions of the gastrointestinal tract, liver, and kidney. Genes are expressed in a continuum from tissue-specific to organ-specific.

Looking for evidence of biological relevance in these gene clusters, a number of features are immediately apparent. Clusters tend to encapsulate a large number of technical replicates (multiple probe-sets designed to the same gene), and multiple members of the same gene family are frequently observed in the same cluster; nodes representing these probe-sets frequently are directly linked to each other in the networks. Furthermore, analysis of Gene Ontology (GO) [29] descriptions for the genes making up clusters consistently shows significant overrepresentation of gene functions that one would naturally associate with their observed pattern of expression. These associations are known to be coupled with coexpressed (cofunctional) genes [20].

We have been interested previously in the relationship between the tissue specificity of gene expression and the evolutionary origin and function of proteins [30,31]. By dividing mouse “atlas” data up into a number of bins depending on the number of tissues in which they are expressed, we were able to demonstrate that tissue-specific genes, particularly those with signal transduction and transcription factor activity, tend to have evolved later and are more likely to be expressed in a tissue-specific manner. Our ability to explore these relationships further, however, was severely constrained at that time due to limitations of analysis packages available. We therefore sought to revisit this interesting phenomenon. As described previously, genes represented on the GNF1M chip were assigned as belonging to one of four phylogenetic groups depending on our ability to identify common ancestral proteins across species: universal, eukaryotic, metazoan, and mammalian. These data were filtered so as to remove redundant probe-sets detecting expression from the same genetic locus. Having first laid out the nonredundant data and clustered it (*p* = 0.9; MCL inflation 2.2), we were able to interrogate the clusters as to their representation of the phylogenetic age of the genes that composed them (Table 2). The set of genes featured in this network tend to be those that share a high degree of tissue specificity in their expression. As a whole, genes from all four phylogenetic classifications were represented in the graphs but without bias in any classification relative to all genes on the microarray. Calculation of classification biases was performed using an inbuilt Fisher's test of BioLayout *Express* ^3D^ (see Methods), comparing class frequencies in clusters to the graph as a whole. At the tissue level, however, while some clusters of tissue/cell-specific genes showed no bias toward any phylogenetic grouping, others did. A number of the major clusters were clearly enriched with genes that have evolved at different times. Immunologically related gene clusters, particularly those associated with T cell and/or B cell–specific expression and placenta-specific genes, were found to be enriched for mammalian-specific genes. More surprising perhaps was the observation that genes expressed predominantly in the testis were also enriched for mammalian-specific genes. However, evaluation of the functional significance of this observation is limited, as few of these genes have yet to be assigned any functionality or description. In contrast, liver and/or kidney gene clusters showed a significant bias in favour of ancient (universal) genes, many of which appear to encode proteins performing basic metabolic processes that have over time become compartmentalised in the liver and kidney. As such, these data support the evolution of tissue-specific pathways and functions at different times. These observations also suggest that one of the dominant evolutionary pressures on patterns of gene expression is driven by the need to compartmentalise functions of the encoded proteins. The expression of old genes performing functions crucial to survival can become confined to one tissue, as in the case with many liver/kidney-specific proteins, presumably through evolution of new regulatory sequences or by continuing to play a housekeeping role in all cells. Clearly, more recently evolved genes can also evolve to be universally expressed. However, where they encode for proteins involved in functions not present in lower organisms, their expression is more likely to show a tissue-specific expression pattern, as their evolution is tied with the evolution and function of a cell or tissue type.

**Table 2:**
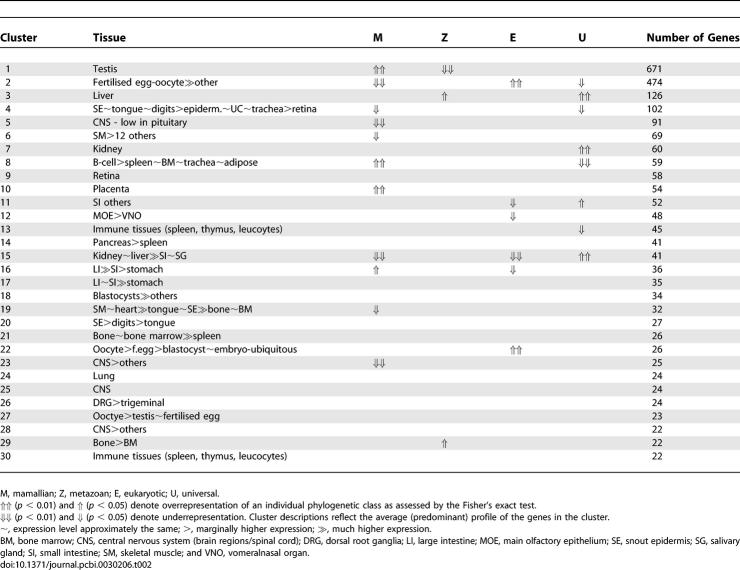
Phylogenetic Analysis of Tissue-Specific Gene Clusters

## Discussion

We believe that this new approach to microarray data analysis and the associated program BioLayout *Express* ^3D^ (see Methods) is a significant advance from previous analytical techniques and tools. Our approach is based entirely on analysis of the coexpression network and uses MCL of the network to rapidly define clusters within that network that accurately capture graph structure. Secondly, our approach is unified and implemented via an open-source software package that generates expression networks from raw expression data, displays the resulting networks interactively in 3-D, integrates data from different sources, and finally provides a platform for clustering, analysis, and data-mining these data. We have used this system to obtain novel insights into gene regulation in the adult mouse protein-coding transcriptome. This analysis has helped illuminate part of the exquisite complexity of gene expression regulation. More data and further questions are required to refine our understanding of the underlying regulatory systems. We believe that BioLayout *Express* ^3D^ will also prove to be a powerful analytical tool when used with other large datasets from a variety of different microarray platforms and experimental paradigms. Together, we believe this system and approach will have wide application to large microarray datasets and beyond.

## Methods

### Expression data and annotations.

The GNF mouse tissue dataset was downloaded as MAS5 normalised files (http://wombat.gnf.org/index.html), and gcRMA data were obtained on request from the GNF. The majority of the data analysis described here is based on MAS5 normalised data from 122 GNF1M chips describing 61 mouse tissues/cell types, each sample type having been analysed in duplicate. All GNF sequences were uniquely mapped (where possible) to Ensembl mouse transcript sequences using SSAHA [32]. Transcripts with the best match above 98% identity and 90% length were taken in each case. The mapped transcripts were then annotated by taking all recorded Ensembl annotations (e.g., GO terms) via the Ensembl Perl API (http://www.ensembl.org/info/software/api_installation.html). Each GO term was mapped onto the GO hierarchy so that terms could be normalised to equivalent levels in the GO hierarchy across sequences. The phylogenetic age of each mouse gene represented on the chip was determined as described previously [31]. These annotations have been used to supplement the annotations provided by the GNF and produced by clustering of the expression data.

Other than setting the Pearson threshold, no other filtration of the data was necessary prior to network construction.

### Network construction and layout.

The BioLayout *Express* ^3D^ JAVA application reads a text file containing expression data in tabular format. Each row of this file starts with a unique probe identifier, followed by a number of columns of annotation data specific to that probe-set. The concluding data columns represent normalised expression values for that probe-set across different microarrays included in the study. The application then performs all-versus-all Pearson correlation calculations for all probes. This step is highly optimised and performed in memory, as the number of calculations required is very large (*P* ^2^/2 pairwise comparisons, where *P* is the number of probe-sets). Pairs of probes whose Pearson correlation is greater than a threshold (*p* ≥ 0.7) are stored in memory and then written to a binary file so that they need not be recomputed for subsequent analysis. The network now consists of probes (nodes) connected by expression correlations above the set threshold (edges).

A new version of the weighted Fruchterman-Rheingold algorithm [25] that has been specifically optimised for large graphs performs a 3-D layout of the network. This layout seeks to keep highly connected nodes in close proximity while minimising the number of edge crossings. A total of 60 iterations using a standard temperature function generally result in good layouts of even large graphs (<10,000 nodes) in a reasonable time (<30 min). The resulting layout may also be stored as a file so that it need not be performed again for subsequent analysis of the same graph.

### Network properties and topology analysis.

Node degree is defined as the total number of edges that connect to a given node. Average node degree is the average degree over all nodes in the network. Network diameter is defined as the longest optimal path between any pair of nodes in the network. Clustering coefficient is a measure of small-world properties of the graph [33], and average eccentricity is defined as the average length of optimal paths between all pairs of nodes. The randomisation of the graph was performed as described elsewhere [34]. Two edges of the graph are picked randomly, and, given certain conditions, one vertex of the first edge is switched with one vertex of the second edge. This has been applied a thousand times for each edge on all the edges of the graph. The resulting graph conserves the same degree distribution as the original graph. The IntAct graph uses all the human protein interactions available in the IntAct database as of August 2006 [35]. The Lehner-Fraser protein interaction graph uses a set of human predicted interactions based on orthology with fruitfly, worm, and yeast [36]. The STRING graph uses human protein interactions from version 6.3 of the STRING database [37].

### Expression landscape plot.

A landscape plot is an alternative representation of a dendrogram in which it is easier to comprehend the component structure at a given threshold as implied by the tree structure. First, the order of the leave nodes (genes) is determined by recursively ordering the subtrees at all internal nodes, starting at the root node. The subtrees at a given internal node are reordered such that the larger tree occurs on the left. Subsequently, a histogram is created on all the joining events corresponding with the pairs of subsequent genes in the ordered list. This number of events is one less than the number of genes. The height of a joining event is simply the Pearson threshold at which the two genes become part of the same component.

### Visualisation engine.

The Java OpenGL (JOGL) library is significantly faster than native Java2D rendering. The network is rendered using optimised OpenGL display lists that make use of hardware acceleration available on most modern graphics cards. This results in an interactive 3-D representation of the network that is fast enough for very large graphs. The limiting step here is generally the memory storage requirements for large graphs and not the rendering itself. In the final 3-D representation, nodes are displayed as spheres or points connected by edges coloured according to their correlation. The user can interact with the representation by zooming, scaling, rotation, translation, and selection of the graph. A built-in annotation and expression viewer allows groups of selected nodes to be compared in terms of their coexpression and annotations. Using the annotation viewer, it is possible to colour the network according to annotation and hide/show nodes that possess specific annotation(s). Whilst improvements in the layout and visualisation capabilities of BioLayout *Express* ^3D^ have been specifically tailored toward large expression graphs, analysis of networks derived from other data sources is also considerably enhanced.

### Graph clustering.

We use the MCL algorithm [27] to cluster this network according to connectivity and local structure. The MCL algorithm [27] is designed specifically for the clustering of simple or weighted graphs. It has previously been used in many fields, including computational graph clustering, detection of protein families from similarity graphs [7], and the isolation of functional modules from protein–protein interaction graphs [28]. BioLayout *Express* ^3D^ calls MCL, passes the current network and clustering parameters, and receives back from MCL a list of nodes and their cluster assignments. These cluster assignments are added to the network as annotation data and provide a basis for statistical analysis of annotation terms across clusters. MCL exploits the observation that the number of “higher-length” (longer) paths between two arbitrary nodes in a natural cluster is high. In particular, this number should be high, relative to node pairs lying in *different* natural clusters. The MCL algorithm finds cluster structure in graphs by a mathematical bootstrapping procedure. The process deterministically computes (the probabilities of) random walks through the graph, and uses two operators transforming one set of probabilities into another. It does so using the language of stochastic matrices (also called Markov matrices) that captures the mathematical concept of random walks on a graph.

The MCL algorithm simulates random walks within a graph by alternation of two operators called *expansion* and *inflation*. Expansion coincides with taking the power of a stochastic matrix using the normal matrix product (i.e., matrix squaring). Inflation corresponds with taking the Hadamard power of a matrix (taking powers entrywise), followed by a scaling step, such that the resulting matrix is stochastic again, i.e., the matrix elements (on each column) correspond to probability values.

### Statistical analysis of clusters.

When annotated data is used to generate a clustered network, it is possible to locate annotations present within a cluster that occur more frequently than one would expect by chance. This has been shown to work extremely well for commonly used descriptive terms such as those provided by GO [29], pathway membership, although BioLayout *Express* ^3D^ allows such analysis for any annotation terms assigned to nodes. Frequencies of annotation terms assigned to the original data are stored in memory and used as control frequencies. The frequency of terms within a cluster are then calculated, and overrepresentation is calculated as:

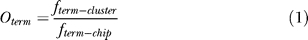



The significance of the observed overrepresentations is then examined by performing a large number (1,000) of random control experiments in which the same number of randomly selected genes are examined for overrepresentation. The standard deviation and mean of these random trials allows us to calculate a *Z*-score for terms within a cluster, where *Z* > 2.0 may be deemed significant.

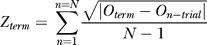



GO terms were flattened to a specific level by first counting for each term how many steps were required (minimum) to get to the root of the tree for that term. A specific annotation term could then be flattened to lower levels by moving up the tree to the desired level. Annotation terms that were already below the desired level were discarded.

### Fisher's test.

To get another estimate of the statistical significance of a term in a cluster, we also implemented the two-sided Fisher's exact test in a similar way to other methods (e.g., GoMiner [38], a widely accepted and used tool for microarray functional analysis). The appropriateness of the Fisher's test in this context has been stated previously, with the proviso that it be used judiciously [38]. A Bonferroni correction is used to correct Fisher's *p*-values for multiple testing. For pragmatic reasons, we combined all three measures into an overall score using a simple function. Relative entropy is used as a direct proportional measure, whereas Fisher's *p*-values and the member count are combined in a discrete-valued function. This modulation function is a product of an empiric quality measure (1 is best, 0.01 is worst) for the Fisher's *p*-values and the member count, respectively. The final overall score is the product of relative entropy and the modulation value. The resulting scoring system can be used for ranking large annotation lists. The final decision is made by a human expert, but the system suggests and presents highly relevant terms

### Software availability.

BioLayout is written in Java and is available as an executable .jar file from http://www.biolayout.org. BioLayout *Express* ^3D^ is released under the GNU Public License. The MCL algorithm is available separately also under GNU Public License (http://micans.org/mcl). BioLayout *Express* ^3D^ uses the Java OpenGL library libraries that are included in the .jar file, but are platform-specific. Hence, the current version of BioLayout *Express* ^3D^ is only available for Macintosh, Linux, Windows, and Solaris. To use BioLayout *Express* ^3D^ effectively for rendering large graphs, a 3-D hardware accelerated graphics card and 1 GB of RAM are recommended.

## Supporting Information

Figure S1 Effect of Adding Noise to Edges on Graph Clustering (18 KB PDF)

## Abbreviations

GNF: Genomics Institute of the Novartis Research Foundation
GO: Gene Ontology
MCL: Markov cluster
