# Supplementary figures and images for: Construction, Visualisation, and Clustering of Transcription Networks from Microarray Expression Data
Source: PLoS Comput Biol. 2007 Oct 26;3(10):e206. doi: 10.1371/journal.pcbi.0030206 (PMC2041979; doi:10.1371/journal.pcbi.0030206)

**Effect of adding noise to edges on graph clustering**

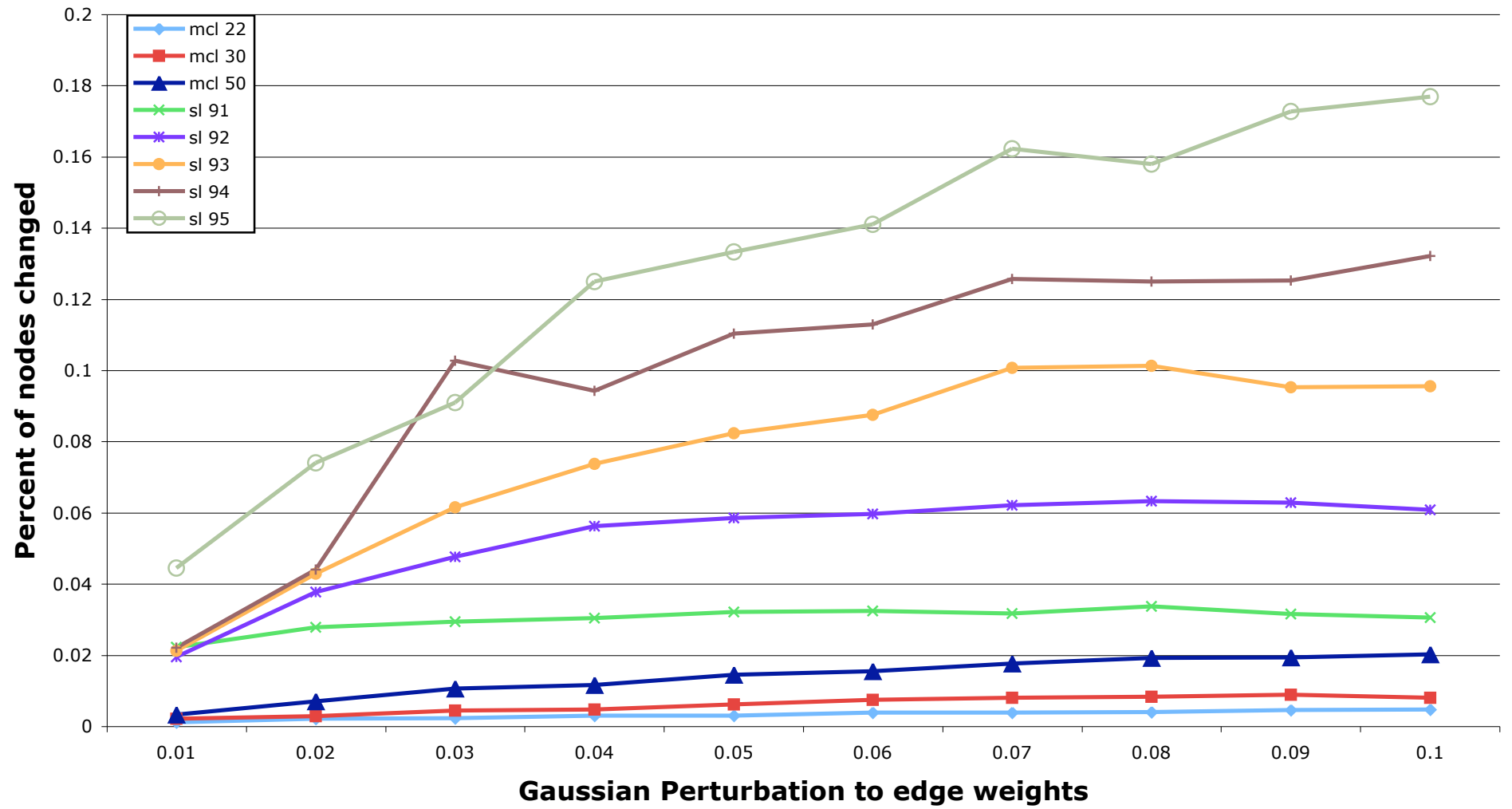

Supplement: Figure S1 — (18 KB PDF) [file pcbi.0030206.sg001.pdf]
